# Biliary Calculi

**Published:** 1869-11

**Authors:** 


ARTICLE II.—*Biliary Calculi.*

THE subjoined sketch of a case of biliary calculi may prove of practical value to some of the readers of the JOURNAL, who may hereafter be called upon to treat this painful and dangerous malady.

Mr. D., 415 S. Morgan street, about fifty years of age, had suffered, during many years, with recurrent attacks of violent pain "in his stomach," so severe in their character as on one or two occasions to prostrate him in the street. Within the past year, the attacks have been more frequent, but less severe. He came under my professional care about three months ago, at which time his condition was as follows: Figure spare and thin; countenance pale, but presenting none of the evidences of cachexia; skin in a healthy condition; respiration and circulation normal; appetite moderately good; had vomited very rarely a clear, watery fluid; bowels constipated habitually; fœces clay-colored, waxy and tough; urine normal in quantity, but high colored.

The pain, which had been occasional was now continuous, and was confined to the right hypochondrium, and accompanied with considerable tenderness; it was aggravated about an hour or two after each meal, from which time it gradually diminished.

Considering the case one of sub-acute gastro-duodenitis, involving partially the ductus communis choledocus, directed half a grain of proto-chloride of mercury every four hours, until five grains had been taken; to be followed by ten grains of sub-carbonate of Bismuth, before each meal. This was followed by such marked relief that the patient considered himself well, and I saw no more of him for two months.

About four weeks ago, the pain returned with redoubled violence. The same course of treatment was directed, but ineffectually. The iodide of silver, in solution, was followed by an aggravation of the symptoms so decided that the patient discontinued its use, after having taken two or three doses. By this time the pain had changed its locality to the left hypochondrium, and its character to a regularly intermittent paroxysmal type, simulating gastralgia very closely. Iron (Quevenne's) 3 grains, and strychnia $\frac{1}{40}$, together with ten grains sub-carbonate of bismuth, every 6 hours, was then administered for two days, without producing the slightest amelioration of the symptoms. A review of the case at this point left the diagnosis somewhat obscure. Believing the position assumed as the basis of treatment to have been clearly determined, the results of treatment indicated that it was at best but a secondary lesion. What was the primary one?

Of this problem there were three possible solutions. Schirrous of the pylorus gastralgia, and biliary calculi. While the case presented symptoms common to each and all of these conditions, it likewise manifested others quite as contradictory. As an *experimentum crucis*, the patient was directed to take one pint of pure olive oil at a single dose. This was followed within a few hours by free purgation, and the discharge of *fourteen biliary calculi*, varying in dimensions from the size of a pea to that of a grape. The evacuations now resumed their normal color.

The removal of this *fons et origo mali*, left the patient entirely free from suffering, beyond what was occasioned by some slight tenderness in the right hypochondrium.

It is due to a highly esteemed professional friend to state, that the remedy was suggested in the course of an informal conversation, some months since, by Dr. Ira Hatch of this city. Having tested it experimentally, on two occasions, in his own practice, with as complete success, he is fairly entitled to the claim of originality, until the right of priority is clearly established by another.

W. H.

NO. 188 S. SANGAMON STREET,
*November* 4, 1869.

